# Three-Dimensional Porous Copper-Graphene Heterostructures with Durability and High Heat Dissipation Performance

**DOI:** 10.1038/srep12710

**Published:** 2015-08-03

**Authors:** Hokyun Rho, Seungmin Lee, Sukang Bae, Tae-Wook Kim, Dong Su Lee, Hyun Jung Lee, Jun Yeon Hwang, Tak Jeong, Sungmin Kim, Jun-Seok Ha, Sang Hyun Lee

**Affiliations:** 1 Institute of Advanced Composite Materials, Korea Institute of Science and Technology, Joellabuk-do 565-905, Republic of Korea; 2 Department of Advanced Chemicals & Engineering, Chonnam National University, Gwangju 500-757, Republic of Korea; 3 Department of Nanomaterials and Nano Science, Korea University of Science and Technology (UST), Daejeon 305-350, Republic of Korea; 4 Korea Photonics Technology Institute, Gwangju 500-779, Republic of Korea; 5 School of Polymer Science and Engineering, Chonnam National University, Gwangju 500-757, Republic of Korea; 6 Optoelectronics Convergence Research Center, Chonnam National University, Gwangju 500-757, Republic of Korea

## Abstract

Porous materials have historically been of interest for a wide range of applications in thermal management, for example, in heat exchangers and thermal barriers. Rapid progress in electronic and optoelectronic technology necessitates more efficient spreading and dissipation of the heat generated in these devices, calling for the development of new thermal management materials. Here, we report an effective technique for the synthesis of porous Cu-graphene heterostructures with pores of about 30 μm and a porosity of 35%. Graphene layers were grown on the surfaces of porous Cu, which was formed via the coalescence of molten Cu microparticles. The surface passivation with graphene layers resulted in a thermal conductivity higher than that of porous Cu, especially at high temperatures (approximately 40% at 1173 K). The improved heat dissipation properties of the porous structures were demonstrated by analysis of the thermal resistance and temperature distribution of LED chips mounted on the structures. The effective combination of the structural and material properties of porous Cu-graphene heterostructures provides a new material for effective thermal management of high-power electronic and optoelectronic devices.

Effective thermal management is a crucial requirement for better performance and functionality of electronic and optoelectronic devices^1^,^2^. The heat flux generated during the operation of a device is strongly linked to its efficiency, lifetime, and failure^3^. Therefore, removal of the generated heat is important, requiring methods for heat transfer and dissipation from the device. For example, although only a small amount of power is required to operate a single LED, large heat fluxes exist in LED chips driven at high currents because of the ambient temperature and joule heating at the contacts and the p-n junctions^4^. In microprocessors, overheating of the electronic components results in performance degradation and failure of a system. For high-performance devices and future integration of multiple microprocessor functions, much greater dissipation of the generated heat fluxes will be required^5^,^6^.

Porous structures are very attractive for thermal management purposes as heat transfer or blocking components in diverse applications^7^,^8^. The large surface areas of the pores offer the possibility for substantial increases in heat transfer to another medium, such as a gas or liquid. Therefore, the geometries and surfaces of the porous structures with opened or closed pores are important factors that determine their thermal properties.

From a material perspective, nanostructured carbon materials, including nanodiamonds, nanotubes and graphene, are promising candidates for thermal management because of their superior thermal conductivities^9^. Graphene and graphene composites, in particular, has advantages in process compatibility with electronic devices^10^,^11^,^12^,^13^. However, the extraordinary thermal properties of graphene are restricted by its high anisotropy, which results in a cross-plane thermal conductivity that is two orders smaller than the in-plane value. To overcome this limitation, 3D porous graphene or graphene-based heterostructures have been suggested as ideal materials for thermal transport as well as for electrodes in energy devices^14^.

In this paper, we report a strategy for the synthesis of a 3D microporous graphene-copper heterostructure using chemical vapor deposition (CVD) and sintering of copper microparticles. Single or multilayer graphene was grown on the surface of porous copper to create networks. The porous structures with graphene networks offered better heat conduction, and their large surface areas increased the dissipation of the heat outside the structure. The novel graphene-copper composite showed good performance as a heat sink for high-power LEDs. Therefore, the structure may be utilized for effective thermal management in high-performance electronic and optoelectronic devices with long operating lifetimes.

## Results

### Fabrication of porous composites

**Figure 1:**
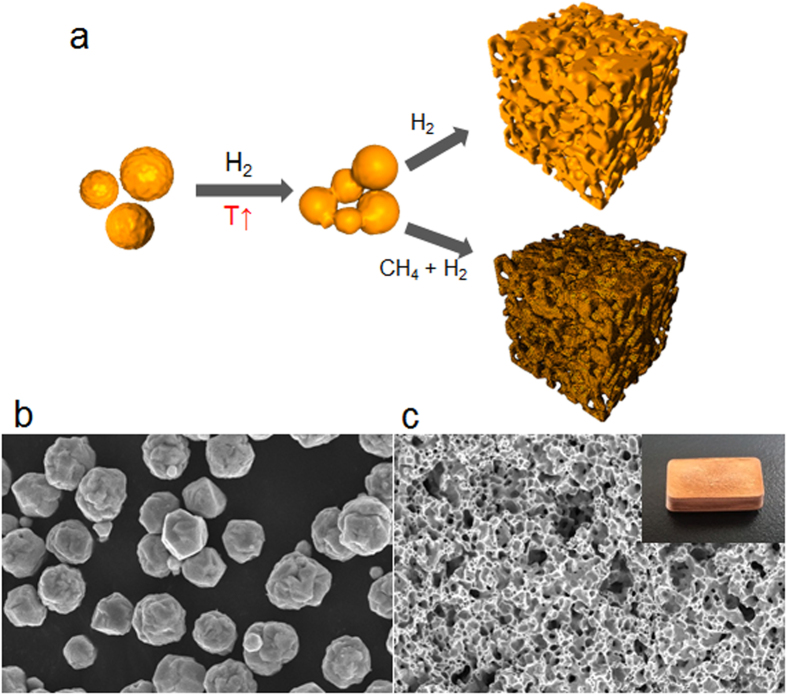
Synthesis of the porous heterostructure using microparticles. (**a**) Schematic of the transformation from microparticles to porous structures. SEM images of (**b**) copper microparticles and (**c**) the porous structure. Scale bars, 10 μm (**b**) and 100 μm (**c**). The inset in Fig. 1(c) shows a typical photo of the porous structure. (width × length × height, 1 × 2 × 0.5 cm^3^).

The porous structures were synthesized by sintering Cu microparticles, and graphene layers were grown on the surface of Cu via CH_4_ decomposition on copper, as described in detail in the Methods section. Figure 1a illustrates the transformation of the copper microparticles to the porous copper (p-Cu) or porous graphene/copper (p-G/Cu) structures. As the temperature increases, the contact points between the Cu particles that are under the greatest pressure begin to deform, and they bond and combine at temperatures below the melting point of copper. The surface melting of Cu microparticles causes them to gradually conjoin with neighboring particles, resulting in porous structures^15^. After formation of the porous structure, CH_4_ gas was introduced as a carbon source to grow the graphene layer on the surface of the p-Cu at 1000 °C. The scanning electron microscopy (SEM) images in Fig. 1b,c show the morphology of the Cu microparticles and the porous structures. The microparticles, which had an average size of 5 μm, formed the porous structures after the thermal treatment. The average pore size in the porous structures is approximately 30 μm, and the porosity is determined to be approximately 35% by using a mercury porosimetry. The carbon source did not influence the morphology of the porous structures, but the surface color was slightly brighter than that of the pure copper porous structure. We confirmed that all the surfaces of p-Cu are coated by graphene by removing the porous Cu structure with a chemical etchant. After etching, the remaining graphene shells are black (Fig. S1 in the Supplementary information). Graphene coated porous Cu showed better chemical durability in strong oxidizer. The surface color of p-Cu became dark in an instant by oxidation, while the brightness of p-G/Cu remained owing to surface protection by graphene (see Fig. S2 in the Supplementary information).

### Structural properties of the porous structures

**Figure 2:**
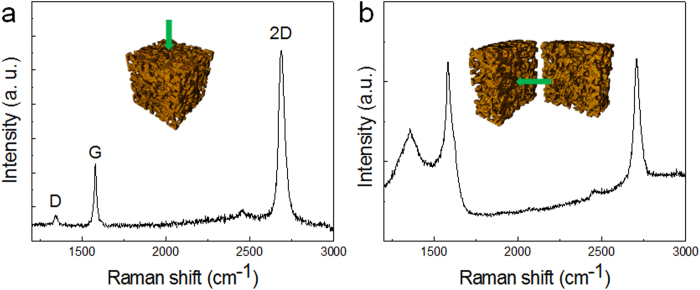
Raman spectra of the graphitic layers grown on the porous structure. Raman spectrum recorded from (**a**) the outer surface of the as-grown sample and (**b**) the cross-section after cutting.

Raman spectroscopy was performed to confirm the existence of graphene layers on the surface, including the surface of the internal pores for samples synthesized under the CH_4_ environment. Figure 2 shows the Raman spectrum obtained from the outer surface and the cross-section of the porous structure under 532-nm laser excitation. The typical graphene Raman peaks (G-band at ~1342 cm^−2^, D-band at ~1580 cm^−2^, and two-dimensional (2D) modes at ~2680 cm^−2^) appear at both locations^16^,^17^. Among the peaks, the result from the outer surface of the sample shows similar features to high-quality graphene grown on Cu foil under the same growth conditions (Fig. S3 in the Supplementary information). The peak for the D band is weak, and the 2D/G peak intensity ratio is approximately 2–2.5, indicating that most of the graphene is present in a single layer. However, in the case of the cross-section of the porous structure, a notably higher intensity for the D band and a lower 2D/G ratio are observed.

**Figure 3:**
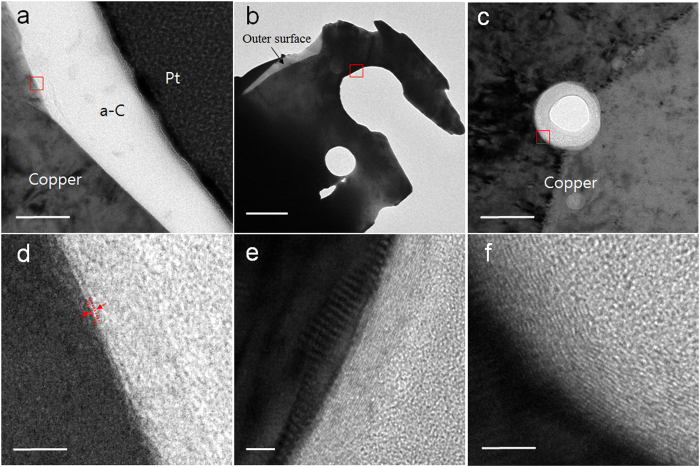
TEM images of graphene layers on the surfaces of a porous structure. TEM images of the outer surface (**a**) and internal micro- (**b**) and nanopores (**c**). (**d**–**f**) High-magnification images from the red boxes in (**a**–**c**) of p-G/Cu. Scale bars, 100 nm (**a,c**), 2 μm (**b**), and 5 nm (**d**–**f**).

Detailed structural analysis of the graphene layers grown on the outer and internal surface of the p-G/Cu structure was performed using transmission electron microscopy (TEM). It was confirmed that few-layered graphene continuously covered the outer surface, as shown in Fig. 3a,d. Figure 3b,c show the micro- and nanopores formed inside the porous structures. Graphitic layers in the range of 5–10 nm thick were observed on the surfaces of the pores, as shown in Fig. 3e,f. In addition, an amorphous carbon layer appears as the pore size decreases (Fig. 3f). The intense D band from the cross-section of the porous structure (Fig. 2b) originated from the highly defective graphitic layers. The structural difference in the graphitic layers between the outer surface and inside the pores may be attributed to different growth kinetics. On the outer surface, the growth of the graphene is governed by a surface reaction, which is similar to the behavior of graphene growth on copper foil. The absorption/desorption of the carbon species onto copper occurs spontaneously in the surface reaction region under low pressure at high temperature^18^. However, in the micro- and nanopores, the injected gas flow is impeded by the complicated structure, and the local pressure increases as the temperature increases. These conditions can possibly lead to bulk gas-phase reactions in the stagnant gas, forming graphitic layers on the surface of the catalyst with a high defect density. The carbon layers far from the copper surface are more defective owing to the absence of the catalytic effect.

### Thermal properties of the porous structures

**Figure 4:**
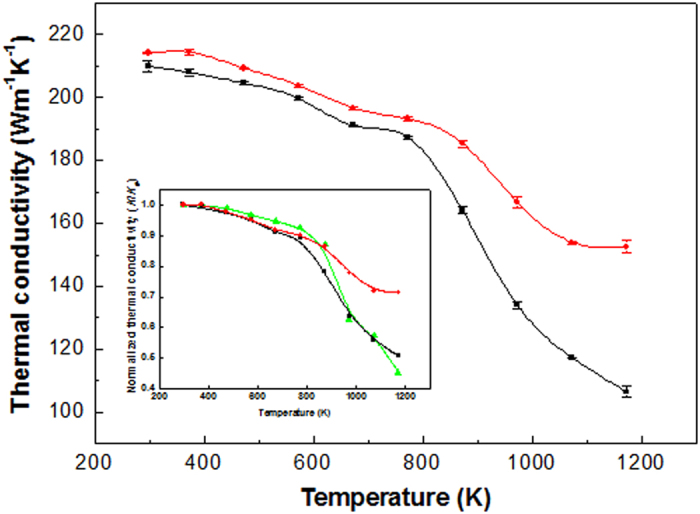
Thermal conductivity, k, of the p-Cu and the p-G/Cu heterostructure as a function of the temperature. The inset shows the plot of the normalized thermal conductivity as a function of temperature; green, high-purity dense copper; black, p-Cu; red, p-G/Cu.

The thermal diffusivity, *κ*, of each of the porous structures was measured using the laser-flash method as a function of specimen temperature over the range from 298 K to 1173 K. Typical thermal conductivities of the samples are shown in Fig. 4 and were determined from *k = κρC*_*p*_, where *ρ* denotes density and *C*_*p*_ denotes specific heat capacity at constant pressure^19^. At room temperature, the *k* values of the p-Cu and p-G/Cu heterostructures were calculated to be 209 Wm^−1^K^−1^ and 214 Wm^−1^K^−1^, respectively. However, the values are smaller than bulk or thin film copper (300–400 Wm^−1^K^−1^)^20^,^21^ due to the pores in the structures. The gas (or vacuum) with low thermal conductivity filling the void space reduces the effective heat-carrying cross-section area. At temperatures below 800 K, the *k* value of the p-G/Cu heterostructure is slightly higher than that of p-Cu. However, above 800 K, the difference between the *k* values for both of the structures increases. The *k* of p-G/Cu is approximately 40% higher than that of p-Cu at 1173 K. The rapid degradation of *k* of p-Cu is probably associated with the formation of cuprous oxide (*k* ~4.5 Wm^−1^K^−1^) between copper and the residual oxygen in the measurement chamber^22^. It has been reported that surface oxidation generally occurs regardless of the ambient oxygen pressure above 873 K^23^. Three factors may explain the higher *k* value in the copper-graphene heterostructures in the high-temperature regime: (1) surface passivation of copper by graphene, (2) enhancement of the phonon contribution and (3) the radiation effect. It is well known that graphene is an excellent gas barrier^24^,^25^. First, the graphene layers coated on the surface of p-Cu play a role in suppressing the surface diffusion of oxygen into copper. Second, regarding the influence of the phonon on *k* at high temperatures, the *k* of pure metal typically decreases with increasing temperature because of a reduction in the electron mean free path (*λ*_*e*_) due to scattering. However, the mean free path (*λ*_*ph*_) of phonons, which are the major heat carriers in graphene, is not significantly reduced at high temperatures^26^. Similar degradation behavior of *k* was observed in high-purity dense copper, as shown in the inset of Fig. 4. Although solid contact conduction predominates in conducting materials, the contribution of radiation becomes substantial at high temperatures. The higher emissivity of graphene (~2.3%) compared with that of Cu (0.01–0.07%) may contribute a larger amount of radiation in the high-temperature region^27^. It is also remarkable that the porous structures with 3D graphene networks show identical thermal properties, independent of the bulk measurement direction.

**Figure 5:**
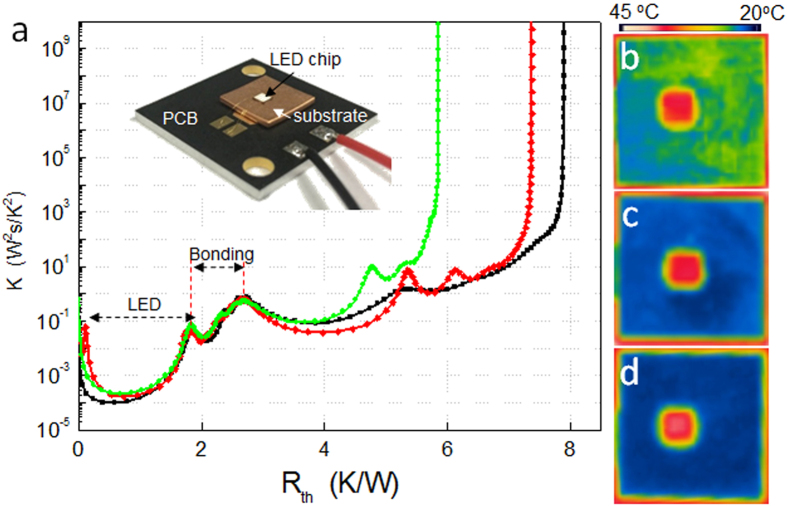
Heat dissipation of LED chips. (**a**) Thermal resistance measurement of LED mounted on three substrates: dense copper (black), p-Cu (red), and p-G/Cu (green). The inset shows a photo of the test. (**b**–**d**) Infrared thermal images of the LEDs on each substrate.

This structure could potentially be applied to thermal management for optoelectronic devices. To compare the heat dissipation of these materials, high-power vertical LED chips (2 × 2 mm^2^) were mounted on three substrates, as shown in the inset of Fig. 5(a): high-purity dense Cu, p-Cu, and p-G/Cu structures. Figure 5(a) shows the results of the thermal resistance analysis for each of the samples at an injection current of 350 mA. The thermal resistances between the LED chips and the submount substrates were found to be 7.91 (dense Cu) 7.38 (p-Cu), and 5.85 K/W (p-G/Cu). To confirm the heat dissipation, we directly visualized the temperature of the LEDs on the substrates using an IR camera (Fig. 5 (b–d)) after operating at 350 mA for 1 hour at room temperature. For the three cases, the emitting sources are the same, but the temperature distributions on the substrates are clearly different. The average temperatures for the Cu, p-Cu, and p-G/Cu substrates (LEDs) were 28.6 (40.9), 21.8 (40.2), and 21.4 (39.0) °C, respectively. Although the difference in the ratio of the temperature for each case does not completely correspond to the results of the thermal resistance, the order of the results is the same.

## Discussion

The p-G/Cu heterostructures were formed via carbon diffusion/nucleation while sintering the Cu microparticles. The graphene layers on the surface of the pores provided better thermal properties, higher thermal conductivities and lower degradation at high temperatures. Heat transfer in the porous materials occurs through three pathways: (1) conduction through solid contacts in the porous structures, (2) convective effects within the pores, and (3) radiation at the surface. Thermal conductivity is the dominant heat transfer mechanism in porous metals and decreases with increasing porosity^28^,^29^. However, we demonstrated that the p-G/Cu heterostructure increased the dissipation of the heat generated by the high-power LED chips, effectively resulting in a lower thermal resistivity of the LED packaging. We also confirmed that the p-G/Cu heterostructure had good heat dissipation properties by comparing the temperature after cooling from identical temperatures for the same amount of time (Fig. S4 in the Supplementary information). The heat transfer mechanism to the outside remains unclear, but it is expected that this heat transfer will become more active in the pores covered by graphene layers. Recently, Al-Mumen *et al.* demonstrated that surface heat convection occurred in micropatterned bilayer graphene at mild temperatures (70 °C)^30^. Our simulation results based on the cellular automaton energy-transport model support the finding that higher convective heat transfer occurs in the p-G/Cu structure than bulk Cu (Fig. S5 in the Supplementary information). In addition, although insignificant, the thermal radiation due to the high emissivity of graphene with a large surface area is likely to contribute to the increase in heat dissipation ability^31^.

In summary, we synthesized Cu and graphene heterostructures with micropores using Cu microparticles. The synthesized graphene layers covered the surfaces of the porous Cu. The combination of the structural and material properties enabled improvement of the durability and heat dissipation properties. The weight reduction of approximately 35% due to porosity is also advantageous for various applications, such as portable electronics and high-power lighting, which require lightweight materials Therefore, we envision that the porous heterostructures will present numerous opportunities for many applications in electronic/optoelectronic devices as well as heat exchangers, filters, and electrochemical cells.

## Methods

### Synthesis of porous structures

P-Cu was synthesized by sintering Cu microparticles (5 μm). The microparticles were loaded in a quartz boat and placed in the center of a horizontal quartz tube. The temperature was gradually increased to 1000 °C for 50 min and held for 20 min under an H_2_ atmosphere (1 mTorr). To grow graphene on the Cu structures, CH_4_ gas was used as the carbon source and introduced into the reactor chamber for 30 min. After the growth, the CH_4_ gas was replaced with H_2_, and the furnace was naturally cooled to room temperature. To compare the thermal properties, commercial high-purity (99.99%) 1-inch Cu was purchased from Taewon Scientific Co. (Korea).

### Characterizations

The morphologies of the synthesized porous structures were characterized using an SEM system (Nova NanoSEM 450, FEI, and 200 kV FE-TEM (JEM-2100F HR, JEOL Ltd)). The open porosity and pore size were measured using a mercury porosimetry ((AutoPore IV 9500, Micromeritics, USA). The Raman spectra were acquired on a Jobin-Yvon LabRam HR 800 micro-Raman system. The temperature-dependent thermal diffusivity and the specific heat were measured using the laser flash method (LFA 457, Netzsch) and a differential scanning calorimeter (DSC 200F3, Netzsch), respectively. The thermal transient measurements of the LED chips mounted on porous structures with silver paste were performed in a thermal transient tester (T3Ster, MicRed). The thermal images and point temperatures of the samples were obtained using an infrared thermal imaging camera (FLIR T-335, FLIR systems, spectral range 7.5–13 μm).

## Additional Information

**How to cite this article**: Rho, H. *et al.* Three-Dimensional Porous Copper-Graphene Heterostructures with Durability and High Heat Dissipation Performance. *Sci. Rep.* **5**, 12710; doi: 10.1038/srep12710 (2015).

## Supplementary Material

Supplementary Information
