# Supplementary material for: Three-Dimensional Porous Copper-Graphene Heterostructures with Durability and High Heat Dissipation Performance
Source: Sci Rep. 2015 Aug 3;5:12710. doi: 10.1038/srep12710 (PMC4522598; doi:10.1038/srep12710)
Supplement: Supplementary Information [file srep12710-s1.doc]

**<Supplementary information>**

**Three-Dimensional Porous Copper-Graphene Heterostructures with Durability and High Heat Dissipation Performance**

Ho-Kyun Rho^1,2^, Seongmin Lee^1^, Sukang Bae^1^, Tae-Wook Kim^1,3^, Dong Su Lee^1,3^, Hyun Jung Lee^1,†^, Jun Yeon Hwang^1^, Tak Jeong^4^, Sungmin Kim^2^, Jun-Seok Ha^2^,*, Sang Hyun Lee^1,3^,*

^1^Institute of Advanced Composite Materials, Korea Institute of Science and Technology, Joellabuk-do 565-905, Republic of Korea
^2^Chonnam National University, Gwangju 500-757, Republic of Korea ^3^Department of Nanomaterials and Nano Science, Korea University of Science and Technology (UST), Daejeon 305-350, Republic of Korea
^4^Korea Photonics Technology Institute, Gwangju 500-779, Republic of Korea
† Present address: BioNano Health Guard Research Center, Daejeon 305-806, Republic of Korea

**
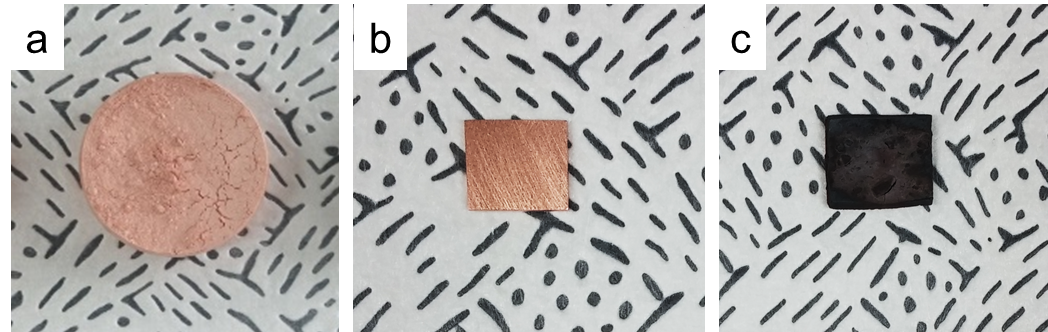
**

**Figure S1| Photographs of porous graphene/Cu (p-G/Cu) heterostructure structure.** (a) as-grown one inch p-G/Cu sample, (b) after cutting and polishing process to 1 x 1 cm^2^ mechanically, and (c) after chemical etching of Cu in ammonium persulfate solution via removing surface graphene using RIE.


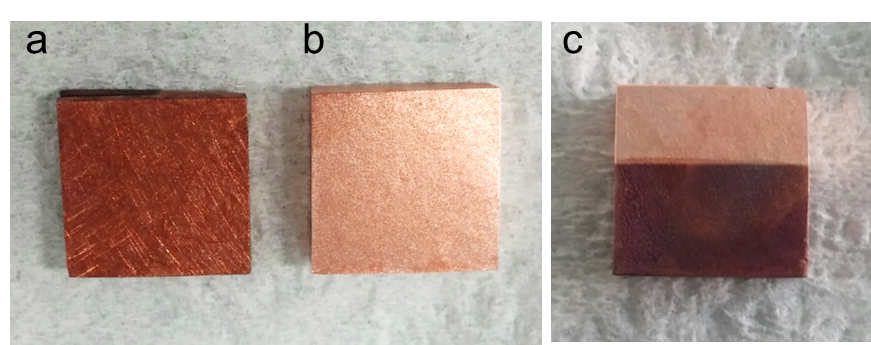


**Figure S2 | The photograph of p-Cu (a and c) and p-G/Cu (b) after dipping in oxidizer.** The samples are dipped into 0.5wt% SeO_2_ solution for 10 sec. The photograph in (c) is p-Cu after dipping a half of sample into the oxidizer as a reference.

**
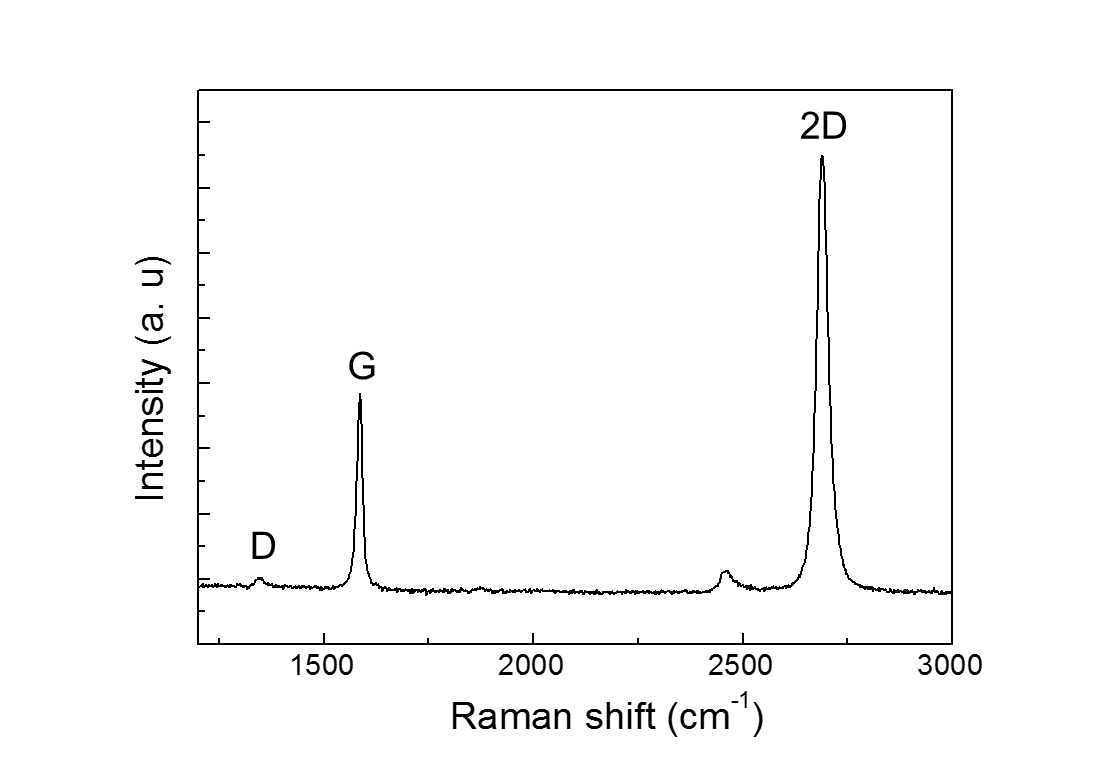
**

**Figure S3 | Raman spectrum of graphene grown on copper foil.** The graphene was grown under same conditions for p-G/Cu described in manuscript and transferred onto SiO_2_/Si substrate.


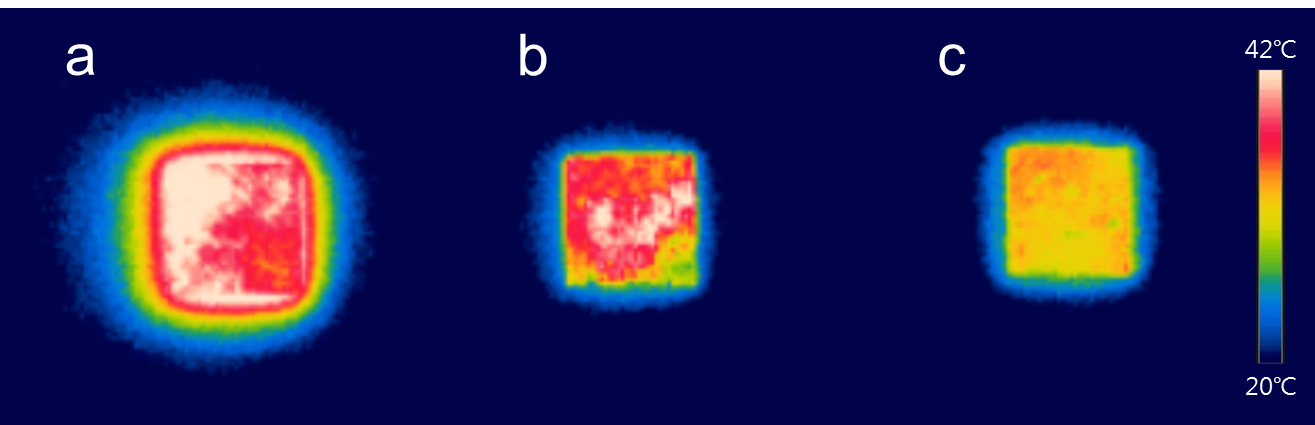


**Figure S4 | Infrared thermal images of heated samples** **after naturally cool down.** (a) pure Cu, (b) p-Cu, and (c) p-G/Cu. All square shaped sample (10 mm(width) × 10 mm(lenth) × 1mm (height)) was putted in the oven at 100 ^o^C for overnight to make same temperature condition.

The samples which were taken out from the oven and naturally cooled down at room temperature. The thermal images were recoded from the sample after cooling for 30s.


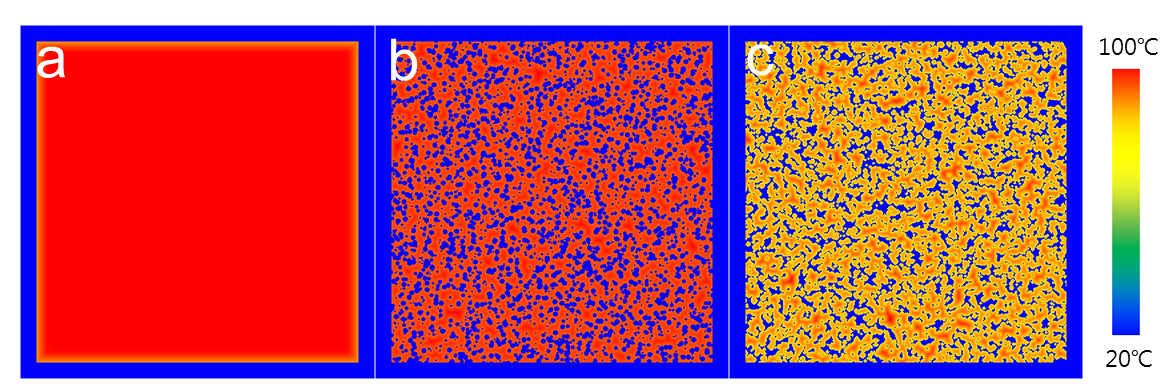


**Figure S5 | Simulated temperature distribution by convection heat transfer based on cellular energy-transport model.** (a) Cu, (b) p-Cu, and (c) p-G/Cu with saturated at100 °C was naturally cooled down for same time at room temperature (20°C).

Analysis model consists of three domains including air, graphene layer, and copper. Heat transferred by convection can be calculated using the equation; *Q = H_c_ × A × ΔT/d* × *t* , where Q is the provided heat, *Hc* is the convection heat transfer coefficient, *A* is the cross sectional area, *T* is the temperature difference, and *d* is the thickness of material. And the taken time (*t*) is regarded as 1 because the analysis method developed in this study is done iteratively using a non-dimensional discrete time interval.
